# A Molecular Dynamics Simulation of Polymers’ Interactions with Kaolinite (010) Surfaces in Saline Solutions

**DOI:** 10.3390/polym14183851

**Published:** 2022-09-15

**Authors:** Gonzalo R. Quezada, Williams Leiva, Jorge H. Saavedra, Pedro Robles, Edelmira Gálvez, Ricardo I. Jeldres

**Affiliations:** 1 Departamento de Ingeniería Química, Universidad de Concepción, Concepción 4030000, Chile; 2 Department of Wood Engineering, Universidad del Bío-Bío, P.O. Box 5-C, Concepción 4030000, Chile; 3 Faculty of Engineering and Architecture, Universidad Arturo Prat, Iquique 1100000, Chile; 4 Escuela de Ingeniería Química, Pontificia Universidad Católica de Valparaíso, Valparaíso 2340000, Chile; 5 Department of Metallurgical and Mining Engineering, North Catholic University, Angamos Av. 0610, Antofagasta 1270709, Chile; 6 Departamento de Ingeniería Química y Procesos de Minerales, Facultad de Ingeniería, Universidad de Antofagasta, Antofagasta 1240000, Chile

**Keywords:** flocculant adsorption, edge surface, kaolinite, molecular dynamic, saline water

## Abstract

The search for polymers that meet the demands of the water recovery process in mining is a contingent challenge. Both the presence of clays and saline waters can impair water recovery from tailings when conventional flocculants are used. In this work, the adsorption of polyacrylamide (PAM), hydrolyzed polyacrylamide (HPAM), poly(2-acrylamido-2-methyl-1-propane sulfonic acid) (PAMPS), polyacrylic acid (PAA), polyethylene oxide (PEO), and guar gum (GUAR) on a kaolinite surface (010) was investigated using classical molecular dynamics. The results show that the presence of sodium chloride modifies the affinities of the polymers with kaolinite (010). At low salt concentrations, the PAM and GUAR polymers generally show higher adsorption due to the formation of hydrogen bridges. However, the highest adsorptions occur in salt solutions in the presence of HPAM by cationic bridging with sodium ions as a mediator. This high affinity of HPAM is not efficient for flocculation because it re-disperses the particles, but it is promising for the design of new additives produced by grafting HPAM groups onto advanced polymers.

## 1. Introduction

Mine tailings generated by the mining industry are an increasingly prevalent topic in social discussions, especially in countries such as Chile, Australia, or Peru, where water scarcity is increasingly problematic, and a large proportion of industrial water is lost in slurries, mainly when these are deposited in tailings storage facilities (TSFs).

The water content retained within the tailings that arrive at TSFs mainly depends on the thickening strategies used. Tailings thickening is the unit stage where flotation tailings are concentrated by sedimentation, generating a clarified water stream that is recovered by the top of the thickener and the underflow of thickened slurry, which is removed by the underflow stream and subsequently transported to the TSF. The underflow concentration depends on mineralogy, water quality, thickening technologies, and chemical reagent management. The chemical reagents used mainly correspond to soluble polymers of high molecular weights, which present functional groups capable of interacting with the surfaces of the dispersed particles, agglomerating them into larger structures that sediment quickly.

The issue is especially complex when clays appear; clays are fine minerals with an atomic structure mainly composed of layers of aluminosilicates, which have various ions present between the layers. This generates a different structural arrangement than minerals such as quartz or other oxides. The layered structure allows clays to be organized in perpendicular plates, generating fractal shapes [1,2]. The most common clays identified in mining deposits are of the kaolin group, kaolinite being the most abundant. This clay has a 1:1 structure in terms of aluminum and silica layers. The aluminum layer is gibbsite with surface hydroxides; the silica layer is siloxane with no hydroxides. The edges are more complex than the faces due to the breakage of the crystals in these planes. It is at the edges where the surface charge is strongly dependent on pH [3]. Their heterogeneous structure, high surface area, and fine size are detrimental to the sedimentation of these particles, even in the presence of flocculants [4]. However, the problem is even more challenging when dealing with a highly saline system [5].

The flocculation of clays in saline systems is a relevant issue from an industrial point of view, considering that the availability of fresh water in the vicinity of reservoirs is decreasing, and there is frequent use of low-quality water, such as seawater [6]. The analysis of these systems is not straightforward, given that many mechanisms act simultaneously. On the one hand, primary particles tend to coagulate in saline media, especially in alkaline conditions, when their surface charge is primarily anionic. In addition, it is common for salts to form cationic bridges between the functional groups of the flocculant and the particle surface, enhancing the adsorption of the polymer. However, it is difficult to predict the modes of adsorption where molecules can lie on the surface, with train configurations reducing the ability to form polymer bridges between particles. Anionic polymers have also been shown to coil in salt solutions, reducing their radius of gyration and thus their ability to bind to several particles simultaneously [7]. For these reasons, it is difficult to anticipate whether a saline medium will be beneficial or detrimental to particle flocculation. Therefore, it is essential to improve our understanding of molecular-scale phenomena, because the phenomena that ultimately determine the macroscopic properties of a suspension and are of industrial interest, such as the sedimentation rate, the quality of water recovered in a thickener, the underflow density, or the rheological properties of the underflow, occur at the molecular level [8,9].

Control possibilities in thickening and tailings management stages are mainly limited to technological aspects of the thickeners and reagent management strategies. Technological changes often generate relevant changes in the parameters of industrial interest; for example, moving from a conventional thickener to a paste thickener can increase the percentage of solids in the underflow slurry by up to 15%, causing a significant increase in the recovery of clarified water. Other, more conservative examples may include changes in equipment parts, such as the types of feedwells, scrapers, etc. However, these decisions are costly and can only be made at certain times. Reagent management may include changes in the flocculant type, dosage, or injection points. It is to be expected that reagent changes will not lead to such radical changes in the efficiency of the operation as when technological changes are developed; however, reagent handling is an activity that can be manipulated very easily. The study by Grabsch et al. is interesting in this regard [10]. They studied the flocculation kinetics of two commercial PAM-based flocculants, Rheomax^®^ DR 1050 and BASF Magnafloc^®^ 336, when applied to a fine calcite suspension; the study confirmed very different responses to variations in the solid’s concentration. The conventional Magnafloc 336 acrylamide/acrylate copolymer was superior at lower solid levels. In comparison, both products gave comparable performances for low dosages at higher solid levels, in which the effective aggregate volume fraction does not significantly impact the aggregate sizes achieved. However, Rheomax DR 1050 consistently produced larger aggregate sizes and better sedimentation rates for higher dosages at solid concentrations of ≥80 kg/m^3^, consistent with a denser aggregate structure. Tanguay et al. [11] subsequently used these kinetic results to model in 3D the potential consequences on feedwell performance, predicting the scope for doubling solids’ throughput under some conditions merely by changing the type of reagent.

The search for new additives has shown that some molecules can efficiently deal with kaolinite, even in highly saline media. One example is chitosan; some interesting works have shown that the presence of salts helps the coagulation–flocculation of kaolinite; however, when PAM groups are grafted, flocculation decreases, possibly due to the neutrality of PAM [12,13]. This suggests that polysaccharide-type polymers may interact with kaolinite and favor its flocculation. For example, studies applied to potash flotation have indicated that guar gum can adsorb to the surface of clays and avoid phenomena detrimental to the recovery of valuable minerals [14]. Other studies have shown how polyethylene oxide (PEO) can adhere to the clay particles [15] and generate relevant changes in the rheological parameters of the suspensions [16,17]. Mpofu et al. [16] found that, at a neutral pH and no salinity, the PEO polymer is a better flocculant than HPAM due to the repulsion present in the anionic polymer. The polyacrylamide sulfonate polymer (PAMPS) increases the size of its active group while maintaining its anionic charge, in the same way that HPAM does. This indicates that the charge density is further externalized from the main chain; however, the sulfonate group generates a lower local charge density than a carboxylic so that it may decrease adsorption. Additionally, the mixing of flocculants by grafting has shown that the bonding of different groups can improve the sedimentation of clays. The work of Zhao et al. [18] indicates that starch-based polymers can vary their adsorption if a specific functional group is grafted; in particular, the sedimentation of clays improves in the presence of cationic polymers over anionic ones. However, they did not evaluate the effect of salinity. Is also important to note that adsorption has been studied from a kinetic modeling perspective at different flocculant dosages [19,20,21]. These finding can measure the coverage of polymers in a surface at different conditions and obtain kinetics and thermodynamics parameters. However, the obtained results are macroscopic, and it is difficult to isolate the main effects.

It is challenging to seek new knowledge about flocculation phenomena using classical laboratory experiments, especially when it comes to experiments as specific as analyzing the adsorption modes of the molecule on the surface of the particles, variations in the size of the flocculant spin, or the type of interactions between the different functional groups. In this sense, computational tools such as classical molecular dynamics (CMD), which allow for the rigorous control of variables and the analysis of effects that are difficult to observe on a real scale, such as those described above, are of interest. Several works demonstrate the advantages of using CMD to study the adsorption of organic molecules on crystalline surfaces [22].

Specifically, there are advanced works on the subject of kaolinite [23]. There is research in MD that shows that kaolinite can adsorb resins from its siloxane surfaces [24]. Moreover, on the hydroxyl surface it shows affinity with PAM [25,26] and also with cationic polymers [27]. Similar work has been undertaken with montmorillonite, where the mixture of chitosan with HPAM can promote aggregation [28]. However, a rigorous analysis of polymers other than HPAM on the surface of kaolinite has not yet been carried out, much less in the presence of salts. Therefore, in order to understand how adsorption occurs with different polymers, and to improve our knowledge of flocculation mechanisms, it is necessary to conduct a study at the atomic scale. In our previous work, we were able to relate the MD adsorption results with experimental sedimentation [29]. Even though the size of these polymers is short compared to the real polymers, the MD simulation can capture the main mechanisms.

This research aims to study the adsorption mechanisms of a single isolated polymer, such as PAM, HPAM, PAMPS, PAA, GUAR, or PEO, on a kaolinite surface and considering its edge (010). The study, developed using CMD, allows us to establish the groups that present higher affinity on the kaolinite surface and, at the same time, the sites of the kaolinite (010) that are most suitable for adsorbing polymers. The effect of salinity on the formation of flocculation mechanisms is particularly interesting. Therefore, a highly saline system with ionic strength is considered, such as seawater, while a low salinity system is used to emulate fresh water. The results obtained in this work will determine which moieties have better affinities with kaolinite surfaces, and could thus be used when designing new additives for mining industry.

## 2. Materials and Methods

### 2.1. Polymers

Six polymers were previously characterized in salt water [30] and subsequently analyzed for their interaction with quartz surfaces [31], a mineral with low surface charge density. The chosen polymers included the neutral polymers polyacrylamide (PAM), polyethylene oxide (PEO), and guar gum (GUAR). Additionally, a polyacrylic acid (PAA) polymer with all its monomers presenting an anionic charge at pH > 6 was completely dissociated. Also included were hydrolyzed polyacrylamide copolymers (HPAM) with 25% PAA monomers and 75% PAM monomers, and a polyacrylamide sulfonate copolymer (PAMPS) with 25% 2-acrylamide-2-methyl-1-propane sulfonic monomers and 75% PAM monomers. Hydrogen atoms or hydroxides were used at the ends to terminate the monomers, and the configuration of the pendant groups was syndiotactic to generate a homogeneous orientation.

### 2.2. Kaolinite Surface

The kaolinite mineral with the (010) face exposed was based on previous studies where it adopted the AC1 configuration [32]. This surface was chosen because it possesses higher reactivity and a topology able to adsorb molecules [33,34,35]. The unit cell used in this work is defined by (a, b, c) = (0.514, 0.740, 8.920) nm^3^ with an angle of 72.2° between the a and b vectors. This cell has 1 SiOH site and 1 Al(OH)_2_ site, the latter with the ability to deprotonate [36]. From the unit cell, it replicated in the three dimensions, generating a 22 × 14 × 1 supercell with a surface area of 108.0 nm^2^ and a thickness of 1.24 nm.

### 2.3. Force Field

The development of the force field of polymers has been detailed in previous works [30,37], mainly restricted to electrostatic potential (RESP) calculations which are performed to describe the electrical topology of polymers. This is conducted using the program R.E.D. III.52 (UFR de Pharmacie, UPJV, Amiens, France) [38] with the help of the program Gaussian 09 (Gaussian, Inc., Pittsburgh, PA, USA) [39]. The program Antechamber (University of California, San Francisco, San Francisco, CA, USA) [40] was used to construct the topology required for the simulation, which describes the necessary constants for each atom of the polymers using the general Amber force field (GAFF). The parameters are tuned using polymers of 12 to 15 monomers to eliminate edge effects in the parameterization of the core monomers. Results of this parameterization are found in the work of Quezada et al. [30].

The (010) surface of kaolinite is parameterized through the CLAYF-MOH force field [32], which corrects the bonds and angles for the Al(OH)_2_ and SiOH groups appearing on the (010) surface. On the other hand, the correction of this force field by the inclusion of pH is carried out according to the work of Kroutil [41], which details how to correct the surface using density functional theory (DFT) calculations. The correction that has been obtained in previous work [36] is the one that will be used in this work.

The ions present in this work are sodium chloride (NaCl), which can be modeled in solution with the methodology presented by Li et al. [42], with parameters set by the ion–oxygen distance. Finally, water was simulated using the SPC/E model [43], which is simple but gives a correct distribution at ambient conditions.

Lorentz–Berthelot mixing rules considered the connections between the molecules and the Lennard-Jones potential.

### 2.4. Initial Setup

The following steps were used to generate the initial configuration to be simulated by molecular dynamics:

• The kaolinite surface with the (010) side exposed to the liquid was placed at one end of the simulation box at z = 0. As such, the simulation box was adjusted to the x and y dimensions while the z direction was extended to obtain a total of 12 nm. This finally generated the system dimension of (Lx, Ly, Lz) = (11.325, 10.01, 12.00) nm^3^ and (α,β,γ) = (90, 90, 72.2).
• The polymer was placed in the void space at an average distance between the surface and its periodic image in the z-direction. The initial configurations of the polymers were previously obtained in simulations in water. The length of the polymers was 48 monomers for the PAM, HPAM, PAMPS, and PAA polymers, 32 monomers for the PEO polymer, and 12 monomers for the GUAR polymer. These values were chosen to generate a similar length between the polymers. The concentration of these polymers was 0.001 M and approximately 5 g/L, which was necessary in the simulation to model the adsorption process.
• The ions needed to neutralize, and the salts were added randomly in the system’s free space, leaving 0.3 nm between the other molecules present. Two concentrations of NaCl were studied: 0.006 M and 0.6 M, to emulate fresh and saline water systems, respectively.
• Water was added from an equilibrated 30 × 30 × 30 nm^3^ configuration at 300 K, which was inserted as blocks over the simulation box. Water molecules overlapping with other atoms in the system were removed. The criterion for overlap is a distance of less than 0.2 nm.

### 2.5. Molecular Simulation

The simulations were performed using the simulation package Gromacs (Uppsala University, Uppsala, Sweden), version 2021.3 [44], with the GPU enabled for parallel calculations. The GAFF force field was transferred from the Amber format to Gromacs using the ACPYPE program (Alan Silva, Hinxton, Cambridge, UK) [45]. Several steps were performed before the production simulations detailed below:

1. Force minimization steps to relax the atoms from their initial positions until a tolerance of less than 10 kJ/mol/nm was achieved. The surface was constrained in its position where only the hydrogen atoms had free movement.
2. A NVT equilibration simulation for a time of 0.1 ns at a temperature of 300 K where only the water molecules moved.
3. A NVT annealing simulation was carried out with the free molecules and ions, and the temperature was increased rapidly from 300 to 450 K in a time of 0.001 ns, then held for 0.5 ns, and then cooled slowly from 450 to 300 K for 0.5 ns.
4. A NPT equilibration simulation was performed for 2 ns at 300 K and 1 bar, where the system matched its velocity and case size for the set temperature and pressure.
5. A NVT production simulation was carried out for 100 ns at 300 K.

The simulations were performed in the box with a periodicity in all three dimensions to avoid edge effects in the liquid phase. The integration step was 1 fs for the previous equilibration stages and 2 fs for the production stage. The treatment of the bonds was performed using the LINCS algorithm [46] and the long electrostatic range treatment was conducted using the particle mesh Ewald method [47]. The cutoff radii for the van der Walls and Coulombic potential calculations were 1.2 nm. The thermostat used for temperature control was the Nose–Hoover [48,49] with a constant of 2.5 ps, and the barostat for pressure control was the semi-isotropic Parrinello–Rahman [50] with a constant of 1 ps semi-isotropic.

Finally, the investigated parameters were six types of polymers with two salt concentrations; five repetitions of each system were performed to improve the statistics of the simulations, giving a total of 60 simulations.

### 2.6. Results Processing

The post-processing programs of Gromacs processed the results obtained from the simulation. The gmx mindist program was used to quantify the surface adsorption, which counts the interactions between a pair of atoms of two selected molecules: in this case, the polymer and the surface. This was used to determine the number of interactions over time and to identify the indices of the atoms that make up the contact. The minimum distance to consider this interaction was 0.5 nm.

A proprietary program was then used to quantify both hydrogen and cation bridges. Both interactions are similar when a positive atom joins two electronegative groups; in hydrogen bridges, it is caused by hydrogen, and in cation bridges by a cation. In the case of hydrogen bridges, a minimum distance of 0.3 nm between the electronegative groups and an angle of 30° between hydrogen–donor–acceptor was considered. In the case of cation bridges, a minimum length of 0.3 nm between the negative groups and the cation was considered. If the cation is bound to both simultaneously, it is considered a cation bridge. Anionic bridges were not considered, because there are no positively charged groups capable of adsorbing anions to form this type of bridge.

The calculation of trains, loops, and tails describes how the polymer adsorbs on the surface, a critical phenomenon in defining flocculation mechanisms. This was achieved by using a proprietary code that measures the distance of the atoms of the main chain to classify them as train, loop, or tail. A minimum length of 0.7 nm between the surface and the central chain atoms was considered in this case.

## 3. Results

### 3.1. Total Polymer Interactions with the Surface

**Figure 1:**
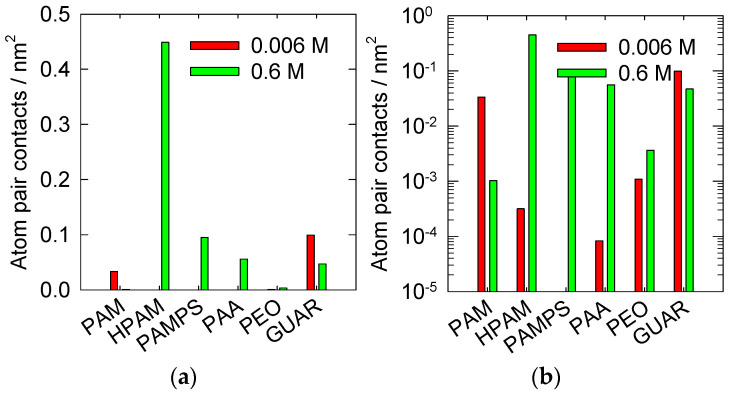
Surface contact between pairs of polymer atoms and the kaolinite surface (010) at pH 7 at 300 K and in the presence of NaCl. (**a**) Linear scale; (**b**) logarithmic scale.

The first results shown in Figure 1 quantify the adsorption of the polymers on the kaolinite surface by calculating the surface interactions averaged over the simulation time. The graphs are offered in both linear and logarithmic scales.

Adsorption at a low salt concentration mainly occurs with PAM and GUAR, with values of 0.04 and 0.1 nm^−2^, respectively. When no salts are present, neutral polymers are more likely to be adsorbed on the surface. However, in the case of PEO, this does not occur, possibly due to its ether groups, which have a greater preference to form bridges with themselves or water [51].

On the other hand, when the salt concentration is high, we can observe different behaviors, as the HPAM polymer presents the highest adsorption on kaolinite with a value of 0.45 nm^−2^. Moreover, it is observed that the PAMPS and PAA polymers show appreciable adsorption of 0.1 and 0.06 nm^−2^, respectively. It is observed that, at high salt concentrations, the observed adsorptions of PAM and GUAR drop.

In general, charged polymers benefit from the presence of salts, and their interaction with the surface increases. This is not the case for the neutral polymers, which showed higher adsorption when the salt concentration was lower and drastically reduced their adsorption with increasing salt concentrations.

**Figure 2:**
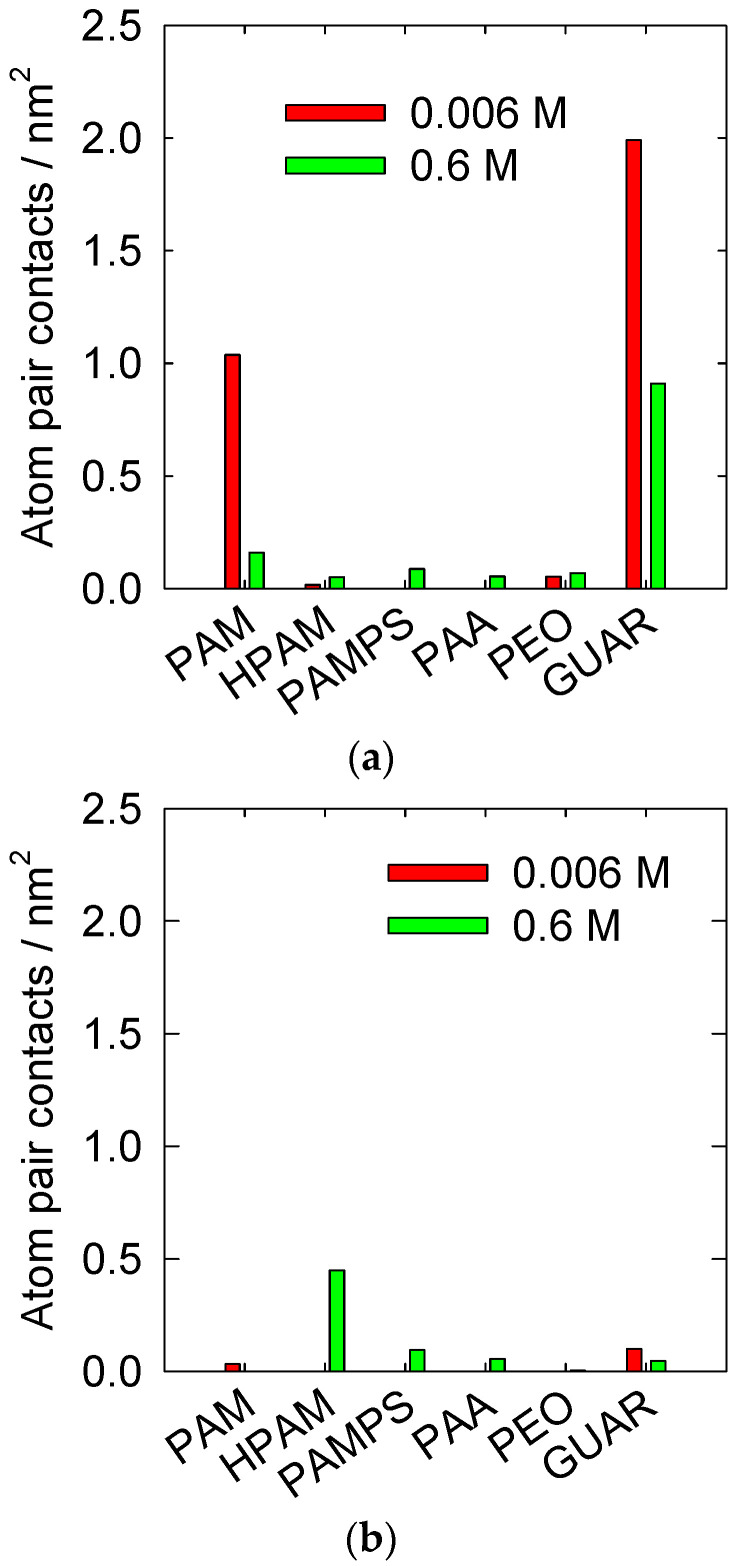
Comparison of quartz (101) and kaolinite surfaces (010) at pH 7 at 300 K. (**a**) Quartz (Reprinted/adapted with permission from Ref. [31]. Copyright (2022), Elsevier) and (**b**) kaolinite (This work).

These results are consistent with what has already been observed in a previous study that analyzed quartz surfaces [31]. Figure 2 compares these results. It is observed that the highest adsorptions occur on the quartz surface with the PAM and GUAR polymers. This significant difference indicates that the quartz surface has a suitable topology for adsorbing PAM and GUAR. In the case of kaolinite (010), the surface behaves differently, allowing slight adsorption with the rest of the polymers, except for HPAM. For HPAM, the adsorption on kaolinite is more significant, a finding consistent with previous results [29]. It can be hypothesized that flocculation is more efficient when the number of interactions is around 0.1 nm^−2^. Using this analysis, we can predict that the PAMPS, PAA, and GUAR polymers may be candidates for the efficient settling of kaolinite in a high-salt-load medium.

### 3.2. Atomic Interactions between the Surface and Polymers

**Figure 3:**
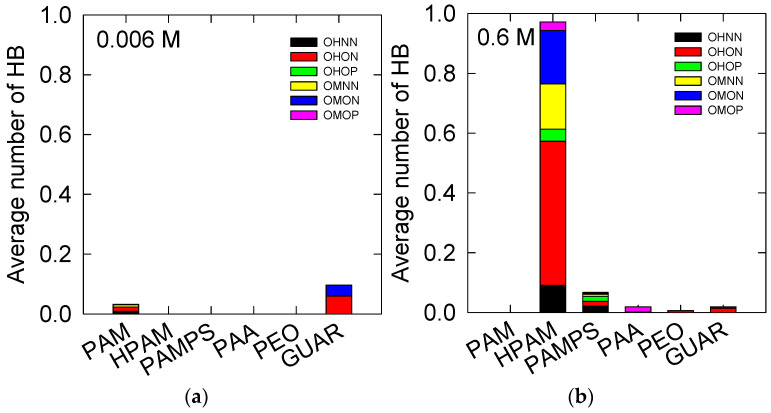
The time average of the HB between the polymers and the kaolinite surface (010). At salt concentrations of (**a**) 0.006 M and (**b**) 0.6 M of NaCl. OH: surface hydroxide, OM: deprotonated surface hydroxide, NN: amine polymer nitrogen, ON: neutral polymer oxygen, OP: deprotonated polymer oxygen.

**Figure 4:**
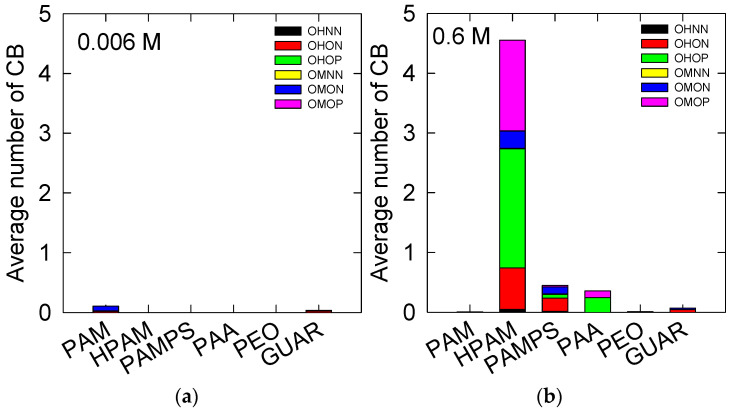
The time average of the HB between the polymers and the kaolinite surface (010). At salt concentrations of (**a**) 0.006 M and (**b**) 0.6 M of NaCl. See Figure 3 for legends.

HB and CB between the polymers and the kaolinite surface were determined, as described in Section 2.6. Figure 3 and Figure 4 show a trend similar to that observed in Figure 1 for both low and high salt concentrations, implying that these interactions are the primary adsorption mechanism of the studied polymers.

At a low salt concentration, we observe that only OHNN-, OHON-, and OMON-type HB interactions are appreciable to PAM. However, their average values are less than ~0.01 in the three cases, so there is no preference among these interactions. In the case of GUAR, there is an average number of hydrogen bridges of 0.1, where 0.06 is from OHON and 0.04 from OMON. The low presence of HB indicates that, even when the salt is not present, the kaolinite surface (010) has little affinity for these polymers.

At 0.6 M NaCl, there is apparent adsorption of HPAM, at higher levels than the rest of the polymers studied. HPAM shows adsorption for all possible combinations of interactions with the surface. The main interaction is OHON, with an average value of 0.5, then OMON with 0.2, and then OMNN with 0.15; the rest account for less than 0.1. OHON is the most frequent because the OH groups on the surface and the ON of the polymer are present in greater quantities. However, the OMON and OMNN interactions indicate that the affinity on charged groups is present and is not repulsive, as occurs on quartz surfaces. This may also happen because of the CB interactions shown in Figure 4, which help to form HBs.

In PAMPS, the most frequent interactions are OHNN, OHON, and OHOP, with values of 0.02, and OMNN, with a value of 0.01. The PAMPS polymer is similar to the HPAM polymer in terms of the number of acrylamide monomers, but their interactions are lower. Thus, the PAMPS charged monomer shows less adsorption than the HPAM loaded monomer. There is very low adsorption for the PAA and GUAR polymers, where these bridges are less than 0.01. For PAA, there are HB interactions, mainly from OMOP. For GUAR and PEO, only HB interactions are observed for OHON, with values of 0.01.

The CBs are presented in Figure 4. The trends, in general, are similar to what has already been shown in HB in Figure 3; both interactions generate a favorable synergy for the interaction of the polymers with the kaolinite surface (010). At low salt concentrations, the only interactions observed are for the PAM and GUAR polymers, with values of 0.1 and 0.05, respectively. For PAM, the primary interaction was OM-Na-ON, while for GUAR, the main interaction was OH-Na-ON.

At high salt concentrations, CB interactions are again in the majority for HPAM, with a total value of 4.5, while PAMPS and PAA have values of 0.5 and 0.4, respectively. When comparing the HB interactions in Figure 3, we notice that CB interactions occur at much higher levels than those obtained by HB. This indicates that the adsorption of polymers on kaolinite is mainly achieved by CB.

The main CBs in HPAM are OH-Na-OP, with a value of 2, and then OM-Na-OP, with a value of 1.5. Other reported interactions are OH-Na-ON, with a value of 0.7, and OM-NA-ON, with a value of 0.3. In the case of PAMPS, the CB interactions are similar to those observed in HPAM, except for the OM-Na-OP interaction, which is absent in PAMPS, where the most frequent is OH-Na-ON, with a value of 0.2. For PAA, it is observed that the main adsorptions are OH-Na-OP, with a value of 0.25, and OM-Na-OP, with a value of 0.1. Finally, the PAM, PEO, and GUAR polymers present minimal appreciable adsorption values lower than 0.05.

Similar results were found in the work of Zeitler et al. [52], where charged kaolinite adsorbed charged molecules with a COO^−^ moiety, such as HPAM or PAA. In their work, the observed interaction is also generated through a cation bond with sodium or calcium ions that mediate the interaction.

### 3.3. Insight of the Adsorption

**Figure 5:**
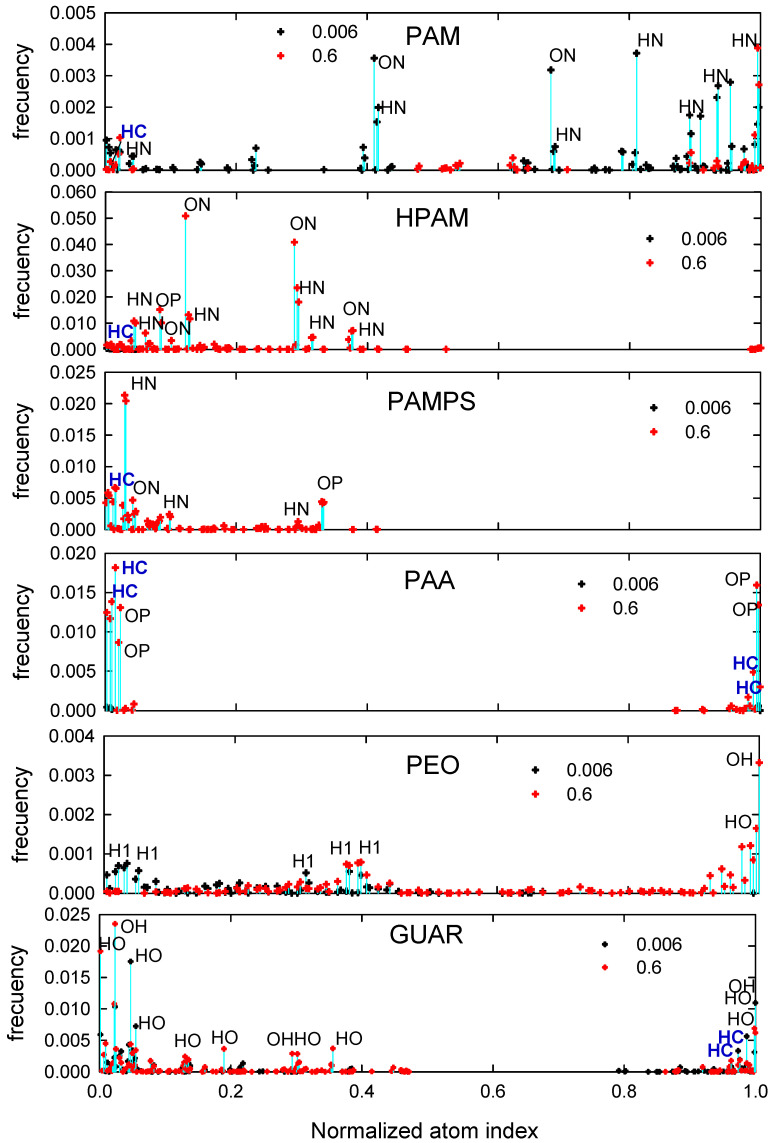
Distribution of interactions of the polymers with the quartz surface at different salt concentrations. HN is amine hydrogen, HC and H1 are carbon-hydrogen, OH is hydroxyl oxygen, and HO is hydroxyl hydrogen.

In this section, the contact distributions between the surface and the polymers are determined, in order to inspect with which atoms and at what chain positions the adsorptions mainly occur. The distributions are presented in Figure 5, where labels of the types of atoms were placed according to the frequency of contacts.

In the case of PAM, it is observed that most of the contacts are for the 0.006 M salt concentration. In this case, the atoms that most contact the surface are ON and HN. It is also possible to find contacts with HC atoms, which correspond to aliphatic hydrogens. For HPAM, the interactions are more significant, and it can be seen that the primary contacts occur with ON atoms, then HN, and some OP and HC. For PAMPS, a higher frequency of HN is observed, and then HC, OP, and ON; here, it is also observed that the interactions occur at the ends of the polymer.

In the case of PAA, the effect of adsorption at the ends of the polymer is more significant; interestingly, HC interactions are relatively frequent, compared to those of the OP type. The PEO polymer presents a more uniform interaction with the surface, showing minor interactions along the chain, and polymer adsorption only at the edge, where the hydroxyl group is the highest. Finally, GUAR has the strongest HO- and then OH-type interaction, which are the hydroxyl groups of the polysaccharide; there is also a tendency to adsorb at the ends of the chain.

The results indicate that interactions by HC atoms are frequent, as seen in previous work [31]. This group acts similarly to a charged group shielded by cations, thus allowing an attraction that is neither HB nor CB, which could be called hydrophobic due to the nature of the polymer atom that binds with the surface.

### 3.4. Conformation of the Polymer on the Quartz Surface

**Figure 6:**
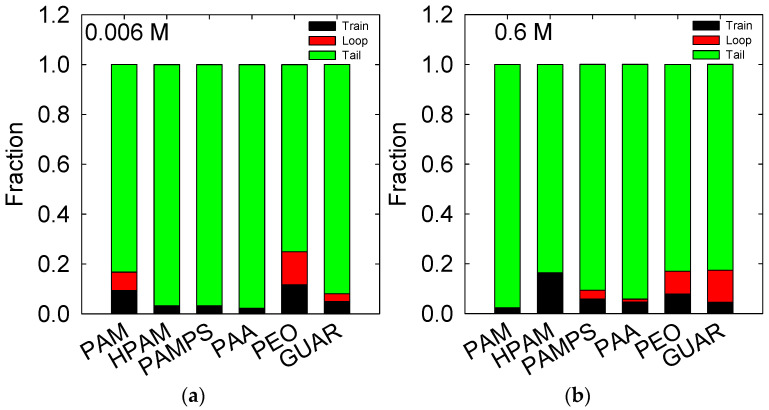
Time average of the train, tail, and loop fractions at salt concentrations of (**a**) 0.006 M and (**b**) 0.6 M of NaCl.

The train, loop, and tail adsorption modes are analyzed to describe polymers’ adsorption phenomena. This is achieved by classifying which monomers of the chain are adsorbed and which are exposed to the aqueous medium. Figure 6 shows the results for both salt concentrations and the different polymers. In all cases, adsorption occurs on a small portion of the polymer’s monomers, where a large portion is exposed as a tail and, to a lesser extent, as a loop.

At low concentrations of salt, PAM and GUAR show adsorptions with portions of loops, indicating that their adsorption occurs on more than one adsorbed site on the surface, in remote positions, to form the loop. The other polymers show only points of adsorption where the monomers are slightly adsorbed, and only PAA shows adsorption with more monomers. These results for HPAM, PAMPS, PAA, and PEO have low representativeness because, according to Figure 1, their adsorption is minimal or practically null.

In the case of 0.6 M salt concentration, we see that the most adsorbed HPAM polymer presents an interaction without loops but with the most significant portion of train (18%). This indicates that its adsorption is strong and continuous on the adsorbed sites. It has been observed that, at pH 11, the formation of the train increases, and the formation of loops also appears [29]. For PAMPS cases, a lower amount of train is observed than for HPAM (~6%), but loops (4%) are present due to the pendant group of PAMPS, which is more extensive. PAA shows a similar presence of trains as PAMPS, but no loops, possibly because its pendant group is small. GUAR shows a high formation of loops (12%) and a low formation of trains (4%), which is due to its larger volumetric size and lower capacity to bend [30]. For PAM and PEO, their behaviors are shown in Figure 5, whereby PAM has low interaction and, therefore, low train formation (~2%); in the case of PEO, there is generalized adsorption along the chain, and this generates a large formation of loops (10%).

The results shows that, when it comes to quartz surfaces, these polymers also present a configuration that is mainly tail, which is favorable to promote aggregation. Nevertheless, these polymers are very short compared to real polymers; therefore, these results indicate that, in the vicinity of 10 nm of the surfaces, they behave mainly as a tail configuration.

## 4. Conclusions

The interactions between PAM, HPAM, PAMPS, PAA, PEO, and GUAR polymers on the surface of kaolinite (010) were analyzed using CMD simulations. Considering industrial concerns in the context of low-quality waters, this study aimed to identify the attributes of conventional polymers that are more likely to work in low- and high-ionic-charge environments.

The results show that kaolinite and its edge surface (010) have a higher affinity with PAM and GUAR polymers at low salt concentrations. HPAM has the highest adsorption at high salt concentrations, followed by PAMPS and PAA. The interactions are mainly of the CB type, which helps the formation of HB interactions by synergy. Some interactions can be called hydrophobic, when the aliphatic hydrogens are the ones that approach the surface.

The conformation of the polymers is mainly concentrated in the tails; in general, more than 70% of the tail is exposed to the medium in all the cases studied. The highest adsorption at low salt concentrations is in PAM and GUAR, with a predominance of loops in the conformation. At high salt concentrations, HPAM is adsorbed in greater quantities on the surface, but it does not form loops, meaning that there is a reasonably strong interaction between HPAM and kaolinite.

These results help to classify the polymers, so that one can infer which functional groups work best according to the selectivity of the polymers. This allows for improved flocculation efficiency depending on the type of water in question.

## Data Availability

The data presented in this study are available on request from author G.R.Q.
